# Chicoric acid binds to two sites and decreases the activity of the YopH bacterial virulence factor

**DOI:** 10.18632/oncotarget.6812

**Published:** 2016-01-01

**Authors:** Alicja Kuban-Jankowska, Kamlesh K. Sahu, Magdalena Gorska, Jack A. Tuszynski, Michal Wozniak

**Affiliations:** ^1^ Department of Medical Chemistry, Medical University of Gdansk, Gdansk, Poland; ^2^ Department of Physics, University of Alberta, Edmonton, Canada; ^3^ Division of Experimental Oncology, Department of Oncology, University of Alberta, Cross Cancer Institute, Edmonton, Canada

**Keywords:** chicoric acid, caffeic acid, chlorogenic acid, protein tyrosine phosphatase YopH, Immunology and Microbiology Section, Immune response, Immunity

## Abstract

Chicoric acid (CA) is a phenolic compound present in dietary supplements with a large spectrum of biological properties reported ranging from antioxidant, to antiviral, to immunostimulatory properties. Due to the fact that chicoric acid promotes phagocytic activity and was reported as an allosteric inhibitor of the PTP1B phosphatase, we examined the effect of CA on YopH phosphatase from pathogenic bacteria, which block phagocytic processes of a host cell. We performed computational studies of chicoric acid binding to YopH as well as validation experiments with recombinant enzymes. In addition, we performed similar studies for caffeic and chlorogenic acids to compare the results. Docking experiments demonstrated that, from the tested compounds, only CA binds to both catalytic and secondary binding sites of YopH. Our experimental results showed that CA reduces activity of recombinant YopH phosphatase from Yersinia enterocolitica and human CD45 phosphatase. The inhibition caused by CA was irreversible and did not induce oxidation of catalytic cysteine. We proposed that inactivation of YopH induced by CA is involved with allosteric inhibition by interacting with essential regions responsible for ligand binding.

## INTRODUCTION

**Figure 1:**
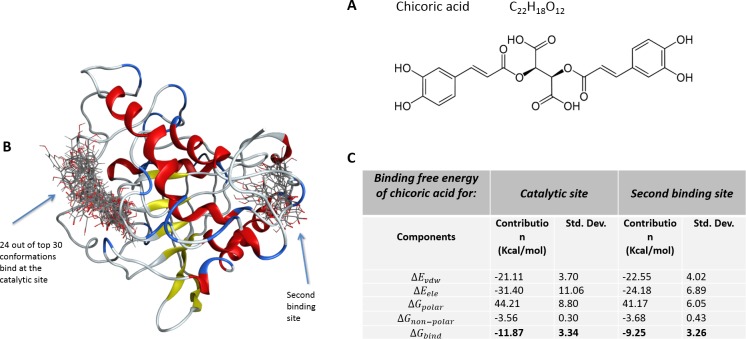
**A**. The structure of chicoric acid; **B**. Top 30 conformations of chicoric acid obtained from blind flexible docking of CA into YopH; **C**. Binding free energy and its components for the YopH-chicoric acid complex by MM/GBSA method (kcal mol-1).

Chicoric acid (CA; Figure 1A), a derivative of both caffeic acid and tartaric acid, known also as cichoric acid and dicaffeoyltartaric acid, is the main phenolic compound found in *Echinacea purpurea* [1]. It was identified in many plant families, including those of seagrass, horsetail, fern and lemon balm [2]. Due to its presence in basil, chicory and lettuce, CA is important ingredient of Mediterranean diet [3, 4, 5]. Chicoric acid is one of the numerous active ingredients (alkamides, polysaccharides, and glycoproteins) associated with human health benefits from *E. purpurea* dietary supplements [6, 7] and compared with other phenolic acids, it succeeds on the nutraceutical market.

Chicoric acid is a valuable natural product of special interest owing to its large spectrum of beneficial biological properties. It has been shown to have immunostimulatory properties, promoting phagocyte activity *in vitro* and *in vivo* [8], and to inhibit hyaluronidase, a key enzyme involved in bacterial infection [9]. In addition, CA has antiviral activity [10] and has been reported to inhibit HIV integrase and replication [11, 12, 13]. The inhibitory properties of CA against HIV-1 integrase were confirmed by computational modeling performed by one of the co-authors of the present paper [14]. The activity of CA against the herpes simplex virus has been demonstrated [15, 16]. The antioxidant activity of CA was found to be comparable with that of rosmarinic acid [17, 18]. The anti-proliferative activity has been shown for CA [3], and anti-cancer, through inducing apoptosis of human colon cancer cells [19]. Chicoric acid, through potent binding at the allosteric site, has been showed to inhibit allosterically protein tyrosine phosphatase PTP1B, which play essential role in diabetes and breast cancer [20, 21]. There are many studies on implications of other PTPs in cancer development [22, 23, 24].

Caffeic acid is one of the main natural phenols present in the argan oil, but it can also be found in coffee or red wine [25]. Caffeic acid has been showed to have anti-inflammatory and antioxidant activity [26]. Inhibitory effect of caffeic acid on cancer cell proliferation has been also reported [27, 28]. Chlorogenic acid is the ester of caffeic acid present, i.a. in potatoes [29], with antioxidant activity [30]. It has been demonstrated that chlorogenic acid may slow the release of glucose into the bloodstream after a meal [31].

*Yersinia* genius contains three species of bacteria pathogenic to humans: plague-causing *Yersinia pestis*, septicemia-inducing *Yesinia pseudotuberculosis* and *Yersinia enterocolitica*, which is responsible for a range of gastrointestinal disorders [32]. *Yersinia pestis* is transmitted by fleas while *Y. pseudotuberculosis* and *Y. enterocolitica* are transmitted by the fecal oral route [33].

*Yersinia* sp. utilizes a type III secretion system for translocation of virulence effectors into the host cell [34]. All three *Yersinia* species contain a 70kb plasmid that encodes the complex type III secretion system and effectors (Yops). During infection, *Yersinia* translocates Yop virulence effectors into a host cell leading to inhibition of the innate immune response [35].

One of Yersinia's outer membrane protein effectors is a highly active YopH protein tyrosine phosphatase, which is essential for virulence since the YopH mutant plasmid is avirulent [36]. YopH is causing deregulation of cellular functions, disrupting focal complex structures and blocking phagocytosis [37]. YopH disturbs the focal adhesions by dephosphorylation of the focal adhesion kinase (FAK) and suppresses the production of reactive oxygen species by macrophages [38]. YopH has a similar amino-acid sequence in the active site as other PTPs [39].

The hallmark defining the classical PTPs is the strictly conserved active site sequence C(X)_5_R within the catalytic domain, which constitutes the phosphate-binding pocket of the enzyme [40]. Like eukaryotic PTPs, YopH catalyzes the hydrolysis of the phosphate moiety on tyrosine residues within a highly conserved binding pocket, which is also characterized by the closure of the WPD loop upon ligand binding [41]. The cysteine residue inside the active site exists in the thiolate anion form, and is highly prone to oxidation [42]. Oxidation of the cysteine residue leads to the formation of a reversible form of the sulfenic acid residue, while a highly oxidizing environment can induce further oxidation yielding physiologically irreversible sulfinic and sulfonic acid residues, all of which consequently cause inactivation of the enzyme [43]. Oxidative stress, defined as excessive reactive oxygen species (ROS) formation, may induce inactivation of protein tyrosine phosphatases. Inactivation *via* oxidation was suggested as a mechanism of protein tyrosine phosphatases regulation [44].

Due to the fact that chicoric acid is promoting phagocytosis [8] and is able to effectively inhibit protein tyrosine phosphatase PTP1B [20], we decided to examine the effect of CA on bacterial tyrosine phosphatase from *Yersinia enterocolitica*. We also compared it with the effect of CA on human CD45 phosphatase. We performed assays with recombinant enzymes as well as computational analysis of chicoric acid binding. In addition, we performed similar studies for caffeic and chlorogenic acids to compare the results.

## RESULTS

### Docking studies shown that chicoric acid can bind to YopH catalytic and secondary binding site

Chicoric acid molecule was docked into the 3D structure of YopH in order to investigate the possible binding conformation and affinity. We performed blind flexible docking and retained top 30 conformations from docking runs. While 24 of these top scoring conformations of chicoric acid bind to the active site of YopH, we found that there are 6 conformations that bind to a second binding site of YopH (Figure 1B). The docking studies showed that chicoric acid can be easily accommodated inside the binding site and binds specifically in a catalytic center of YopH (Figure 1B).

The binding free energy and its components were calculated for the YopH-chicoric acid complex by the MM/GBSA method. The calculated free binding energies as kcal mol^−1^ are presented in Figure 1C. In comparison to the free binding energy for the YopH-natural substrate phosphotyrosine complex previously calculated by our group with the same methods and parameters as −23.63±4.37 kcal mol^−1^ [45], the strength of binding of chicoric acid in YopH is lower (−11.87±3.34 kcal mol^−1^).

### Similar compounds as chlorogenic and caffeic acid are able to bind only in catalytic site of YopH

**Figure 2:**
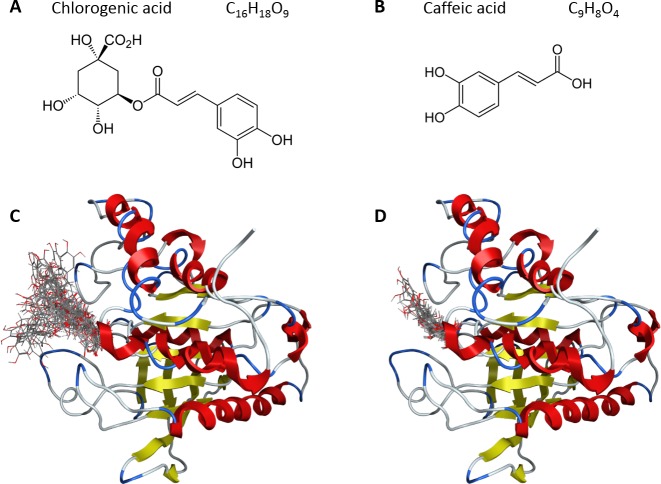
**A**. The structure of chlorogenic acid; **B**. The structure of caffeic acid; **C**. Top 30 conformations obtained from blind flexible docking of chlorogenic acid into YopH; **D**. Top 28 conformations obtained from blind flexible docking of caffeic acid into YopH.

Chlorogenic and caffeic acid molecules were docked into the 3D structure of YopH in order to investigate the possible binding conformation. We performed blind flexible docking and retained top conformations from docking runs. All of these top scoring conformations of chlorogenic (Figure 2A) and caffeic acid (Figure 2B) bind to the active site of YopH. We have not observed any binding to a second binding site of YopH for these compounds.

### Recombinant PTP YopH from Y. *enterocolitica* and human CD45 enzymatic activity inhibition

**Figure 3:**
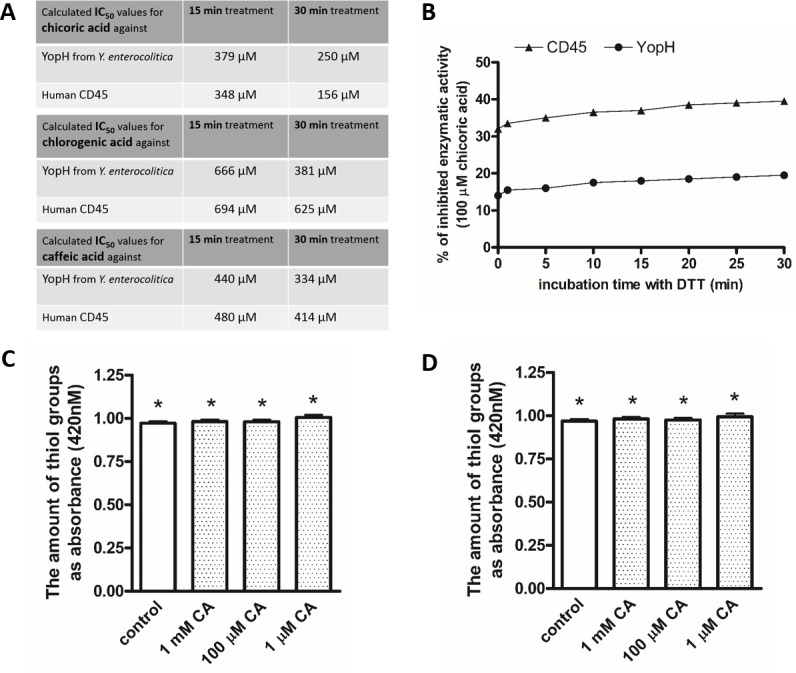
**A**. IC50 values of chicoric, chlorogenic and caffeic acids for YopH and CD45 inhibition. IC50 values were determined from a plot presenting acid concentration versus percentage of the enzymatic activity measured as absorbance with pNPP substrate of recombinant CD45, YopH after 15 and 30 minutes incubation with inhibitor; **B**. Reduction assay of YopH Y. enterocolitica and CD45 activity with DTT. Recombinant YopH and CD45 was pretreated for 15 minutes with 100 μM chicoric acid and subsequently incubated with 10 mM DTT to reverse the inhibition. The percent of inhibitory effect in comparison to original activity of untreated YopH and CD45 was measured every minute on microplate reader as absorbance at 405 nm using pNPP substrate. Data presented as percent of inhibition; **C**. The amount of modified YopH thiol adducts with NBD (Cys-S-NBD adducts) after 15 minutes of treatment with chicoric acid (CA). Data presented as absorbance (420 nm), means±SD (n=3). One-way Anova test. * Means were not significantly different from control (*P* > 0.05); **D**. The amount of modified CD45 thiol adducts with NBD (Cys-S-NBD adducts) after 15 minutes of treatment with chicoric acid. Data presented as absorbance (420 nm), means±SD (n=3). One-way Anova test. * Means were not significantly different from control (*P* > 0.05).

We examined the effect of chicoric acid treatment on bacterial tyrosine phosphatase from *Yersinia enterocolitica* and human CD45 phosphatase. We also compared it with the effect of chlorogenic and caffeic acid. We found that chicoric acid can reduce enzymatic activity of both YopH and CD45 phosphatase. We observed higher inhibitory effect of chicoric acid on YopH and CD45 in comparison to chlorogenic and caffeic acids (Figure 3A). We calculated IC_50_ values from a plot presenting chicoric acid's concentration *versus* percentage of the enzymatic activity measured as absorbance with *p*NPP substrate of recombinant YopH and CD45. The calculated IC_50_ values of chicoric acid are similar for both enzymes and are in the micromolar range (Figure 3A).

### Chicoric acid induces irreversible inactivation of YopH and CD45

We performed a reduction assay to examine the reversibility of chicoric acid induced YopH and CD45 inactivation. The results showed that the inhibition caused by chicoric acid cannot be reversed to YopH and CD45 original activity (Figure 3B). In our studies, the 100 μM chicoric acid caused YopH and CD45 inactivation cannot be restored after a 30-minute incubation with 10 mM dithiotreitol (Figure 3B).

### Mechanism of YopH inhibition caused by chicoric acid

Many studies showed that the enzymatic activity of PTPs can be reduced by oxidation of the catalytic cysteine residue [43, 44, 46], which we have also previously demonstrated for peracids [47]. To study the mechanism of chicoric acid caused inactivation of YopH, we decided to examine the amount of non-oxidized thiols groups. We performed an assay for thiol adducts forming with NBD-Cl. Our results show that YopH and CD45 after treatment with chicoric acid possess the same amount of reduced thiol groups as in untreated control (Figure 3C, 3D). The results allow us to assume that the catalytic cysteine residue did not undergo oxidation after incubation with chicoric acid and that the inactivation caused by chicoric acid is probably not involved with oxidation of catalytic thiolate in active site.

### Docking and molecular dynamic simulations of chicoric acid-YopH complex

**Figure 4:**
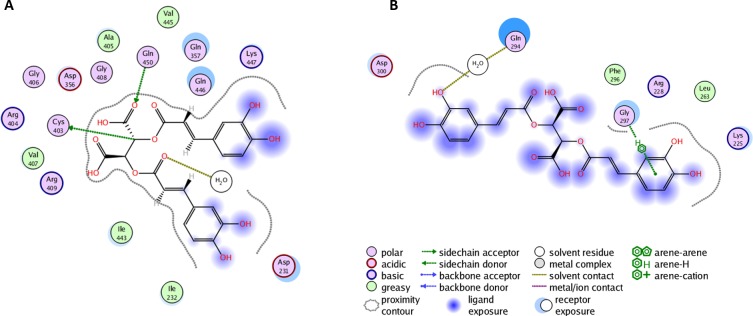
**A**. Molecular dynamics simulation of chicoric acid in the YopH catalytic site. The PLIF diagram for the best binding pose of chicoric acid in the YopH binding site. In predicted binding pose, two carboxyl groups of chicoric acid are directed toward essential Cys403 and Arg409 residues in the active site. There are electrostatic interactions between polar groups of chicoric acid with Cys403, Gln450 and water; **B**. The PLIF diagram for the best binding pose of chicoric acid in YopH obtained from site-specific docking on 286-297 and nearby residues. In predicted binding pose, Gly297 interacts with one aromatic ring of chicoric acid using arene-H interaction. Gln294 interacts using a water molecule. Phe296 and Leu263 are involved in hydrophobic interactions with chicoric acid.

**Figure 5:**
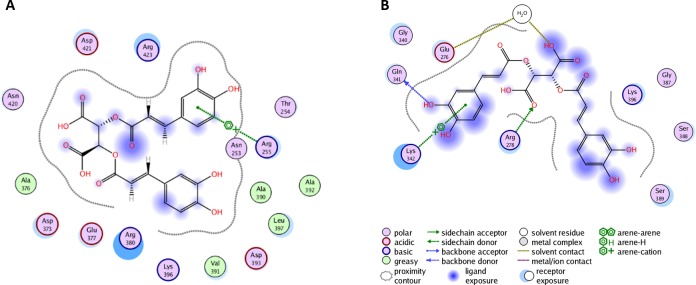
**A**. Docking and interaction analysis of chicoric acid in the YopH second binding site. The PLIF diagram for the best binding pose of chicoric acid in the YopH second binding site. In predicted binding pose, Arg255 interacts with one aromatic ring of chicoric acid using arene-cation interaction. The Arg380 from α4 is observed in close proximity; **B**. The PLIF diagram for best binding pose of chicoric acid in YopH obtained from site-specific docking on 380-392 and nearby residues. Lys342 interacts with one aromatic ring of chicoric acid via an arene-cation interaction. Gln341 and Arg278 also interact with chicoric acid via hydrogen bonds. Glu276 interacts with chicoric acid through a water molecule.

To examine the interaction between chicoric acid and amino acid residues in YopH binding sites we performed molecular dynamics (MD) simulations. Both blind (Figures 4A, 5A) and site specific (Figures 4B, 5B) flexible docking was performed. We decided to focus on the region surrounding the catalytic pocket from the loop L2 and second binding site. The loop L2 is the region surrounding the catalytic pocket and shows correlated closure with the WPD loop [48]. The mobility of the WPD loop plays an important role in the catalytic process of PTPases. Ligand binding significantly reduces the protein flexibility and constrains the WPD loop predominately in the closed form [48].

### Chicoric acid interactions in YopH catalytic site

In a first step, we examined the interaction between chicoric acid and amino acid residues in YopH binding sites with blind flexible docking. The obtained results are presented as PLIF diagrams for the best binding poses of chicoric acid in the YopH catalytic site (Figure 4A). The PLIF diagram presented in Figure 4A shows the predicted binding pose in catalytic site of YopH, where two carboxyl groups of chicoric acid are directed toward essential Cys403 and Arg409 residues. There are electrostatic interactions observed between polar groups of chicoric acid with Cys403, Gln450 and water.

We performed flexible docking with selection of the residues 287-297 form L2 (Figure 4B). We found that, in the best binding pose selected for this region, the one aromatic ring of chicoric acid interacts with Gly297 using arene-H interaction and Gln294 interacts using a water molecule. We can observe that Phe296 and Leu263 are involved in hydrophobic interactions with chicoric acid.

### Chicoric acid interactions in YopH second binding site

The PLIF diagram presented in Figure 4B shows the best predicted binding pose of chicoric acid in the second binding site of YopH performed with blind flexible docking. There are interactions and binding between chicoric acid and amino acids from the loop correlated with the motion of WPD loop (Figure 5A). In the predicted binding pose, Arg255 interacts with one aromatic ring of chicoric acid using an arene-cation interaction. The Arg380 from α4 is observed in close proximity (Figure 5A).

The crystallographic studies of PTP1B with small molecular inhibitors have revealed that the region of helices *α*3 and *α*6 (corresponding to helices *α*4 and *α*7 in YopH) constitutes an allosteric binding site [49]. An ordered *α*4 helix (residues 380-383) is found to correlate with the closure of the WPD loop, and the flexibility of loop L6 (residues 384-392) is highly associated with the WPD loop movements [48].

Based on these findings, we selected residues 380-392 for flexible docking (Figure 5B). Our interaction analysis, after docking, shows that the one aromatic ring of chicoric acid interacts with Lys342 *via* an arene-cation interaction. Gln341 and Arg278 also interact with chicoric acid *via* hydrogen bonds. There are also observed interactions between Glu276 and chicoric acid through a water molecule.

## DISCUSSION

Chicoric acid has been shown to have antiviral activity [10], by inhibiting HIV integrase [11, 12, 13], immunostimulatory properties, promoting phagocyte activity [5]; and to inhibit hyaluronidase, a key enzyme involved in bacterial infection [9]. The inhibitory properties of CA against HIV-1 integrase were confirmed by computational modeling performed by one of the co-authors of the present paper [14]. Here we described antibacterial properties of chicoric acid against *Yersinia* sp. bacteria due to decreasing the activity of YopH virulence factor which is essential for the induction of the infection process.

In the present paper, using both computational modeling and experimental assays we have demonstrated that chicoric acid can bind to two binding sites in the YopH phosphatase enzyme and that the catalytic site is preferred. We found that similar compounds as chlorogenic and caffeic acids are not able to bind into second binding site of YopH. We discovered that chicoric acid can reduce the enzymatic activity of bacterial PTP YopH from *Y. eneterocolitica* and human CD45 phosphatase. Based on calculated IC_50_ values chicoric, chlorogenic and caffeic acids for YopH and CD45 (after 15 and 30 minutes treatment), we observed higher inhibitory effect of chicoric acid on YopH and CD45 in comparison to chlorogenic and caffeic acids. Our results showed that chicoric acid irreversibly inactivates YopH phosphatase. We also found that the inhibition caused by CA is not involved with oxidation of the catalytic cysteine. We propose that chicoric acid induce allosteric inhibition of YopH activity by binding and disturbing the essential region responsible for active conformation.

Chicoric acid was already reported to allosterically inhibit protein tyrosine phosphatase PTP1B [20]. The allosteric inhibition of PTP1B activity is achieved by perturbation along helices *α*3 and *α*6 [49], the corresponding helices *α*4 and *α*7 in YopH are highly correlated with the motion of the WPD loop, providing structural insights to the role of these helices in allosteric inhibition [48].

The finding that the second substrate binding site is correlated with the dynamics of the WPD loop *via* helices *α*4 and *α*7, as well as loop L4, suggests that they are potential allosteric binding sites for the design of novel, selective YopH inhibitors as antibacterial agents [50]. Based on our docking and molecular dynamic simulation results we propose that chicoric acid induces allosteric inhibition of YopH activity by binding along the catalytic pocket and helices *α*4, thus disturbing WPD loop mobility essential for active conformation and ligand binding.

## MATERIALS AND METHODS

### Recombinant PTP YopH and CD45 activity assay

Bacterial recombinant YopH protein tyrosine phosphatase from *Yersinia enterocolitica* was obtained from Calbiochem. Human recombinant CD45 was obtained from Sigma-Aldrich. The solutions of the recombinant PTPs were prepared in 10 mM HEPES buffer pH 7.4. The final concentration of phosphatase in reaction samples was 0.8 μg/mL (10 nM). The YopH and CD45 enzymes were untreated (control) or treated with solution of chicoric, chlorogenic and caffeic acids. The assay was performed in 96-well microplates, and the final volume of each sample was 200 μL. The enzymatic activities of YopH and CD45 were measured using 1 mM chromogenic substrate *para*-nitrophenyl phosphate (*p*NPP) in 10 mM HEPES buffer pH 7.4, at 37°C. Phosphatase hydrolyzed *p*NPP to *para*-nitrophenol and inorganic phosphate. *Para*-nitrophenol is an intensely yellow colored soluble product under alkaline conditions. The increase in absorbance (due to *para*-nitrophenol formation) is linearly proportional to enzymatic activity concentration (with excessive substrate, i.e. zero-order kinetics) and was assessed at 405 nm on a microplate reader Jupiter (Biogenet) using DigiRead Communication Software (Asys Hitech GmbH).

### Reduction assay with DTT

Subsequently, recombinant phosphatase YopH that had been previously inactivated by chicoric acid, was then treated with 10 mM dithiothreitol (DTT), and the samples were incubated at 37°C to reverse the inactivation, if possible. Restoration of CD45 enzymatic activity was measured every minute as an increase of absorbance taken at 405 nm as described above.

### YopH and CD45 thiol adduct assay

The recombinant phosphatase YopH and CD45 was inactivated by chicoric acid and the amount of modified YopH and CD45 thiol adduct with NBD (Cys-S-NBD adduct) was measured after 30 minutes incubation with NDB-Cl (0.6 mM in a 0.5 mL sample) as absorbance at 420 nm with a spectrophotometer.

### Docking studies

The initial structure of YopH was imported from the RCSB protein data bank (www.pdb.org) with code 2YDU.pdb [51]. The structure was minimized using taff.ff forcefield of the Molecular Operating Environment software (MOE, chemical computing group). Chain A of this pdb file contains 306 residues. The ligand was removed from this pdb file and chicoric, chlorogenic and caffeic acids were docked into the structure of YopH. A blind flexible docking simulation was performed, where the binding site was assumed to be the entire protein. The side chains were kept free to move during forcefield refinement. Alpha PMI is the placement method used with default settings (sample per conformation = 10, maximum poses = 250). London dG rescoring was used with Alpha PMI placement. Termination criteria for forcefield refinement were set as gradient = 0.001 and interactions = 500.

### Molecular dynamics simulations

Top scoring poses from docking that interacted with Cys403 were retained for molecular dynamics simulations using amber12. We allowed Leap module of Amber [52] to add missing hydrogen atoms and heavy atoms using the Amber force field (ff10) parameters [53]. To neutralize the charge of the system, we added sodium/chloride ions. The model was immersed in a truncated cubical shell of TIP3P water [54]. A time step of 2 fs and a direct-space non-bonded cutoff of 10 Å were used. After the protein preparation, all systems were minimized to remove the steric clashes that occurred. The systems were then gradually heated from 10 to 300 K over a period of 50 ps and then maintained in the isothermal-isobaric ensemble (NPT) at a target temperature of 300 K and a target pressure of 1 bar using a Langevin thermostat [55, 56] and a Berendsen barostat with a collision frequency of 2 ps and a pressure relaxation time of 1 ps, respectively. We constrained hydrogen bonds using the SHAKE algorithm [57]. We have used the velocity-Verlet algorithm (default algorithm for the Amber MD package) for MD simulations. Particle mesh Ewald (PME) procedure was used to treat long-range electrostatic interactions using default parameters [58]. After bringing the systems at a suitable temperature and pressure of 300 K and 1 bar, respectively and equilibrating the system for 500 ps, the production run was continued for 20 ns in the isothermal-isobaric ensemble at the target temperature of 300 K and target pressure of 1 bar using the same Langevin thermostat and Berendsen barostat. The structures in the trajectories were collected at 10 ps intervals. The analysis of trajectories was performed with the Ptraj module of Amber.

### Binding affinity calculations

For the binding free energy calculations, we used the standard MM/GBSA method [59]. MMPBSA.py python script was used for MM/GBSA calculations [60]. Before the MM/GBSA analysis, all water molecules and the sodium ions were excluded from the trajectory. The dielectric constant used for the solute and surrounding solvent was 1 and 80, respectively. During the analysis of the MM/GBSA trajectory, snapshots were gathered at 10 ps intervals from the last 500 ps of the 20 ns trajectory.

### Statistical analysis

The experiments were performed at least three times. The data were applied and analyzed with GraphPad Prism (GraphPad Software v.4). Statistical analyses were performed using ANOVA combined with Tukey's test or T test combined with Wilcoxon test. The data were expressed as means±SD. Differences between means were considered significant for *P* < 0.05.

## References

[1] Molgaard P, Johnsen S, Christensen P, Cornett C (2003). HPLC method validated for the simultaneous analysis of cichoric acid and alkamides in Echinacea purpurea plants and products. J Agric Food Chem.

[2] Lee J, Scagel CF (2013). Chicoric acid: chemistry, distribution, and production. Front Chem.

[3] Elansary HO, Mahmoud EA (2015). *In vitro* antioxidant and antiproliferative activities of six international basil cultivars. Nat Prod Res.

[4] Saad EM, Madbouly A, Ayoub N, El Nashar RM (2015). Preparation and application of molecularly imprinted polymer for isolation of chicoric acid from Chicorium intybus L. medicinal plant. Anal Chim Acta.

[5] Zlotek U, Swieca M (2015). Elicitation effect of Saccharomyces cerevisiae yeast extract on main health-promoting compounds and antioxidant and anti-inflammatory potential of butter lettuce (Lactuca sativa L.). J Sci Food Agric.

[6] Barnes J, Anderson LA, Gibbons S, Philipson JD (2005). Echinacea species (Echinacea angustifolia (DC.) Hell., Echinacea pallida (Nutt.) Nutt., Echinacea purpurea (L.) Moench): A review of their chemistry, pharmacology and clinical properties. J Pharm Pharmacol.

[7] Lee J, Scagel CF (2010). Chicoric acid levels in commercial basil (Ocimum basilicum) and Echinacea purpurea products. J Funct Foods.

[8] Bauer R, Reminger P, Jurcic K, Wagner H (1989). Influence of Echinacea extracts on phagocytic activity. Z Phytother.

[9] Bauer R, Lawson L.D., Bauer R (1998). Echinacea: biological effects and active principles. Phytomedicines of Europe: chemistry and biological activity.

[10] Pellati F, Benvenuti S, Magro L, Melegari M, Soragni F (2004). Analysis of phenolic compounds and radical scavenging activity of Echinacea spp. J Pharm Biomed Anal.

[11] Charvat TT, Lee DJ, Robinson WE, Chamberlin AR (2006). Design, synthesis, and biological evaluation of chicoric acid analogs as inhibitors of HIVa integrase. Bioorg Med Chem.

[12] Liu C.Z, Abassi BH, Gao M, Murch SJ, Saxena PK (2006). Caffeic acid derivatives production by hairy root cultures of Echinacea purpurea. J Agr Food Chem.

[13] Healy E F, Sanders J, King P. J, Edwards Robinson W (2009). A docking study of L-chicoric acid with HIV-1 integrase. J Mol Graph Model.

[14] Sahu KK, Ravichandran V, Jain PK, Sharma S, Mouryac VK, Agrawal RK (2008). QSAR Analysis of chicoric acid derivatives as HIV-1 integrase inhibitors. Acta Chim Slov.

[15] Binns SE, Hudson J, Merali S, Arnason JT (2002). Antiviral activity of characterized extracts from Echinacea spp. (Heliantheae: Asteraceae) against herpes simplex virus (HSV-I). Planta Med.

[16] Zhang HL, Dai LH, Wu YH, Yu XP, Zhang YY, Guan RF, Liu T, Zhao J (2014). Evaluation of hepatocyteprotective and anti-hepatitis B virus properties of Cichoric acid from Cichorium intybus leaves in cell culture. Biol Pharm Bull.

[17] Dalby-Brown L, Barsett H, Landbo AR, Meyer AS, Molgaard P (2005). Synergistic antioxidative effects of alkamides, caffeic acid derivatives, and polysaccharide fractions from Echinacea purpurea on *in vitro* oxidation of human low-density lipoproteins. J Agr Food Chem.

[18] Grignon-Dubois M, Rezzonico B (2013). The economic potential of beach-cast seagrass - Cymodocea nodosa: a promising renewable source of chicoric acid. Bot Mar.

[19] Tsai YL, Chiu CC, Yi-Fu Chen J, Chan KC, Lin SD (2012). Cytotoxic effects of Echinacea purpurea flower extracts and cichoric acid on human colon cancer cells through induction of apoptosis. J Ethnopharmacol.

[20] Baskaran SK, Goswami N, Selvaraj S, Muthusamy VS, Lakshmi BS (2012). Molecular dynamics approach to probe the allosteric inhibition of PTP1B by chlorogenic and cichoric acid. J Chem Inform Model.

[21] Aceto N, Bentires-Alj M (2012). Targeting protein-tyrosine phosphatases in breast cancer. Oncotarget.

[22] Hoekstra E, Kodach LL, Das AM, Ruela-de-Sousa RR, Ferreira CV, Hardwick JC, van der Woude CJ, Peppelenbosch MP, Ten Hagen TL, Fuhler GM (2015). Low molecular weight protein tyrosine phosphatase (LMWPTP) upregulation mediates malignant potential in colorectal cancer. Oncotarget.

[23] Bourgonje AM, Navis AC, Schepens JT, Verrijp K, Hovestad L, Hilhorst R, Harroch S, Wesseling P, Leenders WP, Hendriks WJ (2014). Intracellular and extracellular domains of protein tyrosine phosphatase PTPRZ-B differentially regulate glioma cell growth and motility. Oncotarget.

[24] Kuban-Jankowska A, Gorska M, Knap N, Cappello F, Wozniak M (2015). Protein tyrosine phosphatases in pathological process. Front Biosci (Landmark Ed).

[25] Charrouf Z, Guillaume D (2007). Phenols and Polyphenols from Argania spinosa. Am J Food Tech.

[26] Olthof MR, Hollman PC, Katan MB (2001). Chlorogenic acid and caffeic acid are absorbed in humans. J Nutr.

[27] Rajendra Prasad N, Karthikeyan A, Karthikeyan S, Reddy BV (2011). Inhibitory effect of caffeic acid on cancer cell proliferation by oxidative mechanism in human HT-1080 fibrosarcoma cell line. Mol Cell Biochem.

[28] Lin HP, Lin CY, Huo C, Hsiao PH, Su LC, Jiang SS, Chan TM, Chang CH, Chen LT, Kung HJ, Wang HD, Chuu CP (2015). Caffeic acid phenethyl ester induced cell cycle arrest and growth inhibition in androgen-independent prostate cancer cells *via* regulation of Skp2, p53, p21Cip1 and p27Kip1. Oncotarget.

[29] Friedman M (1997). Chemistry, Biochemistry, and Dietary Role of Potato Polyphenols. A Review. J Agric Food Chem.

[30] Kweon MH, Hwang HJ, Sung HC (2001). Identification and antioxidant activity of novel chlorogenic acid derivatives from bamboo (Phyllostachys edulis). J Agric Food Chem.

[31] Johnston KL, Clifford MN, Morgan LM (2003). Coffee acutely modifies gastrointestinal hormone secretion and glucose tolerance in humans: glycemic effects of chlorogenic acid and caffeine. Am J Clin Nutr.

[32] Trosky JE, Liverman AD, Orth K (2008). Yersinia outer proteins: Yops. Cell Microbiol.

[33] Achtman M, Morelli G, Zhu P, Wirth T, Diehl I, Kusecek B, Vogler AJ, Wagner DM, Allender CJ, Easterday WR, Chenal-Francisque V, Worsham P, Thomson NR (2004). Microevolution and history of the plague bacillus, Yersinia pestis. Proc Nat Acad Sci USA.

[34] Bahta M, Burke TR (2012). Yersinia pestis and approaches to targeting its outer protein H protein-tyrosine phosphatase (YopH). Curr Med Chem.

[35] Viboud GI, So SS, Ryndak MB, Bliska JB (2003). Proinflammatory signalling stimulated by the type III translocation factor YopB is counteracted by multiple effectors in epithelial cells infected with Yersinia pseudotuberculosis. Mol Microbiol.

[36] Liang F, Huang Z, Lee SY, Liang J, Ivanov MI, Alonso A, Bliska JB, Lawrence DS, Mustelin T, Zhang ZY (2003). Aurintricarboxylic acid blocks *in vitro* and *in vivo* activity of YopH, an essential virulent factor of Yersinia pestis, the agent of plague. J Biol Chem.

[37] Deleuil F, Mogemark L, Francis M.S, Wolf-Watz H, Fällman M (2003). Interaction between the Yersinia protein tyrosine phosphatase YopH and eukaryotic Cas/Fyb is an important virulence mechanism. Cell Microbiol.

[38] Trulzsch K, Sporleder T, Leibiger R, Russmann H, Heesemann J (2008). Yersinia as oral live carrier vaccine: Influence of Yersinia outer proteins (Yops) on the T-cell response. Int J Med Microbiol.

[39] Black DB, Marie-Cardine A, Schraven B, Bliska JB (2000). The Yersinia tyrosine phosphatase YopH targets a novel adhesion-regulated signalling complex in macrophages. Cell Microbiol.

[40] Tabernero L, Aricescu AR, Jones EY, Szedlacsek SE (2008). Protein tyrosine phosphatases: structure-function relationships. FEBS J.

[41] Zhang ZY, Wang Y, Dixon JE (1994). Dissecting the catalytic mechanism of protein-tyrosine phosphatases. Proc Nat Acad Sci USA.

[42] Pagliarini DJ, Robinson FL, Nixon JE (2004). Protein Tyrosine Phosphatases. Encyclopedia of Biological Chemistry.

[43] Ostman A, Frijhoff J, Sandin A, Bohmer F (2011). Regulation of protein tyrosine phosphatases by reversible oxidation. J Biochem.

[44] Persson C, Sjoblom T, Groen A, Kappert K, Engstrom U, Hellman U, Heldin CH, den Hertog J, Finkel T (2003). Oxidant signals and oxidative stress. Curr Opin Cell Biol.

[45] Kuban-Jankowska A, Sahu KK, Niedzialkowski P, Gorska M, Tuszynski JA, Ossowski T, Wozniak M (2015). Redox process is crucial for inhibitory properties of aurintricarboxylic acid against activity of YopH - virulence factor of Yersinia pestis. Oncotarget.

[46] Ross SH, Lindsay Y, Safrany ST, Lorenzo O, Villa F, Toth R, Clague MJ, Downes CP, Leslie NR (2007). Differential redox regulation within the PTP superfamily. Cell Signal.

[47] Kuban-Jankowska A, Gorska M, Tuszynski JA, Churchill CDM, Winter P, Klobukowski M, Wozniak M (2015). Inactivation of protein tyrosine phosphatases by peracids correlates with the hydrocarbon chain length. Cell Physiol Biochem.

[48] Hu X, Stebbins CE (2006). Dynamics of the WPD Loop of the Yersinia Protein Tyrosine Phosphatase. Biophys J.

[49] Wiesmann C, Barr KJ, Kung J, Zhu J, Erlanson DA, Shen W, Fahr BJ, Zhong M, Taylor L, Randal M, McDowell RS, Hansen SK (2004). Allosteric inhibition of protein tyrosine phosphatase 1B. Nat Struct Mol Biol.

[50] Ivanov MI, Stuckey JA, Schubert HL, Saper MA, Bliska JB (2005). Two substrate-targeting sites in the Yersinia protein tyrosine phosphatase co-operate to promote bacterial virulence. Mol Microbiol.

[51] Kim SE, Bahta M, Lountos GT, Ulrich RG, Burke TR, Waugh DS (2011). Isothiazolidinone (IZD) as a phosphoryl mimetic in inhibitors of the Yersinia pestis protein tyrosine phosphatase YopH. Acta Crystallogr D Biol Crystallogr.

[52] Case DA, Cheatham TE, Darden T, Gohlke H, Luo R, Merz KM, Onufriev A, Simmerling C, Wang B, Woods RJ (2005). The Amber biomolecular simulation programs. J Comput Chem.

[53] Lindorff-Larsen K, Piana S, Palmo K, Maragakis P, Klepeis JL, Dror RO, Shaw DE (2010). Improved side-chain torsion potentials for the Amber ff99SB protein force field. Proteins.

[54] Jorgensen WL, Chandrasekhar J, Madura JD, Impey RW, Klein ML (1983). Comparison of simple potential functions for simulating liquid water. J Chem Phys.

[55] Izaguirre JAC, Catarello DP, Wozniak JM, Skeel RD (2001). Langevin stabilization of molecular dynamics. J Chem Phys.

[56] Berendsen HJC, Postma JPM, van Gunsteren WF, DiNola A, Haak JR (1984). Molecular dynamics with coupling to an external bath. J Chem Phys.

[57] Ryckaert JPC, Ciccotti G, Berendsen HJC (1977). Numerical integration of the cartesian equations of motion of a system with constraints: Molecular dynamics of n-alkanes. J Comput Phys.

[58] Darden T, York D, Pedersen L (1993). Particle mesh Ewald-an N-log(N) method for Ewald sums in large systems. J Chem Phys.

[59] Gohlke H, Case DA (2004). Converging free energy estimates: MM-PB(GB)SA studies on the protein-protein complex Ras-Raf. J Comput Chem.

[60] Miller BR, McGee TD, Swails JM, Homeyer N, Gohlke H, Roitberg AE (2012). MMPBSA. py: An Efficient Program for End-State Free Energy Calculations. J Chem Theory Comput.

